# A team-based competition for undergraduate medical students to learn radiology within the virtual world Second Life

**DOI:** 10.1186/s13244-021-01032-3

**Published:** 2021-06-29

**Authors:** Teodoro Rudolphi-Solero, Alberto Jimenez-Zayas, Rocio Lorenzo-Alvarez, Dolores Domínguez-Pinos, Miguel Jose Ruiz-Gomez, Francisco Sendra-Portero

**Affiliations:** 1 Department of Nuclear Medicine, University Hospital Virgen de Las Nieves, Granada, Spain; 2 Department of Radiology and Physical Medicine, School of Medicine, University of Málaga, Málaga, Spain; 3 Department of Emergency and Critical Care, Hospital de La Serranía, Ronda, Spain

**Keywords:** Game-based learning, Virtual worlds, Medical education, Medical students, Radiology

## Abstract

**Background:**

A multi-user competitive game within the virtual world Second Life for undergraduate radiology learning was adapted for team participation. This study aimed to assess student perception, impact on learning, and eventual correlation of game results with post-exposure tests and course grades.

**Methods:**

The game consisted of six weekly stages, dedicated to thoracic, abdominal, and musculoskeletal radiological anatomy and semiology. Participants had several days a week to review self-guided radiology educational content and then complete individual multiple-choice tests and solve team tasks to progress through the game's ranking. Additionally, they completed a cognitive load test, a questionnaire about the experience and a post-exposure knowledge test.

**Results:**

Fifty-two students organised into 13 teams participated in the game and assessed different aspects of the experience with a mean score ≥ 7.8 on a 10-point scale, highlighting the participation of the teacher (9.3 ± 1.1), the educational contents (8.8 ± 1.4) and the usefulness for their education (8.7 ± 1.4). Participants obtained better post-exposure test results (*p* < 0.007) and better course grades (*p* < 0.021) than non-participants did.

**Conclusion:**

A multi-user game adapted to team competition to learn radiology in Second Life was very positively perceived by third-year medical students, who highly valued its content, organisation, and usefulness for their training. Most of the participants agreed that they had collaborated as a team and that playing in competitive environments helps them learn better. The best post-exposure and academic results compared to non-participating students indicate the potential impact of the game on learning.

**Supplementary Information:**

The online version contains supplementary material available at 10.1186/s13244-021-01032-3.

## Keypoints

- Competing as a team in the virtual world Second Life is an engaging and dynamic blended learning method for learning radiology alongside the undergraduate formal course.
- Medical students appreciate the content, organisation, and educational utility of a game such as the League of Rays and find that competing in teams helps them learn better.
- Compared to individual competition, competing in teams has the advantages of promoting collaborative learning and responsibility in collective work.

## Introduction

Radiology education is an important part of the undergraduate medical curriculum, and the use of e-learning in the teaching of radiology in medical schools is on the rise [1]. Therefore, it is important to explore the applicability of innovative technologies and approaches to teaching and the improvements they bring to learning. Digital games have an interesting educational value, as they can engage medical students in their learning and offer them unique insights on the strengths and weaknesses of their knowledge [2]. Game-based learning, with successive rules, rewards, and achievements to motivate medical students, is gaining impact compared to other traditional training techniques [3–6]. Competition is a core element in educational games [7]. Competitive learning techniques improve academic outcomes and can strengthen cooperation among medical students [8]. When teams of students compete with others, learning techniques combine group rewards with individual responsibility and encourage collaboration. Competition and collaboration together have a recognised positive effect on learning [7].

Virtual worlds are three-dimensional spaces reproduced on the computer screen where users interact through a representation of themselves called an avatar, through which the user can move, interact, and communicate with others [9]. There is great interest in research on education in virtual worlds [10], particularly in the possibilities they offer to develop learning games within them [11–13]. Second Life, launched by Linden Research Inc (San Francisco CA. USA) in 2003, is the most active virtual world in education with healthcare professionals [14]. Interesting education experiences have been carried out in Second Life with patients [15–17], physicians [18, 19], and medical students [20–22]. Users can communicate within Second Life via voice and written chat or, alternatively, sending notecards (in-world written messages that remain stored in the inventory of the receiving avatar, recording the date and time of sending and the avatar that created it). The objects in Second Life are composed of primary objects or prims, which can reproduce a Web page on one or more of their faces. This resource can be used as a presentation system for educational content in Second Life, through panels reproducing simple web pages, created from PowerPoint presentations, with backward and forward buttons [23].

In 2011, a virtual space named “The Medical Master Island” was acquired in Second Life to develop educational innovation activities on radiology [24]. Various learning activities with medical students were carried out to explore the feasibility of synchronous teaching sessions and asynchronous tasks [25], evaluate the perception of the students [26], and compare the learning outcomes of seminars held in Second Life and in real life [27]. In 2015, a multi-user competitive game was designed within Second Life, based on self-guided presentations and multi-choice tests. The game, called the League of Rays, forms the basis of this study. It was designed as an individual competition during the four-month Radiology course, taught in the third year of medicine. Several editions have been previously held, with voluntary [28] and compulsory participation. The authors hypothesise that team competition in a learning game like League of Rays can be carried out with good acceptance by students, favouring collaborative work and providing positive learning outcomes. This study aims to explore the participation of student teams, adapting the rules of the League of Rays game, evaluating students' perception, impact on learning and the eventual correlation of game results with mid-term knowledge tests and course grades.

## Materials and methods

### The virtual environment

**Fig. 1:**
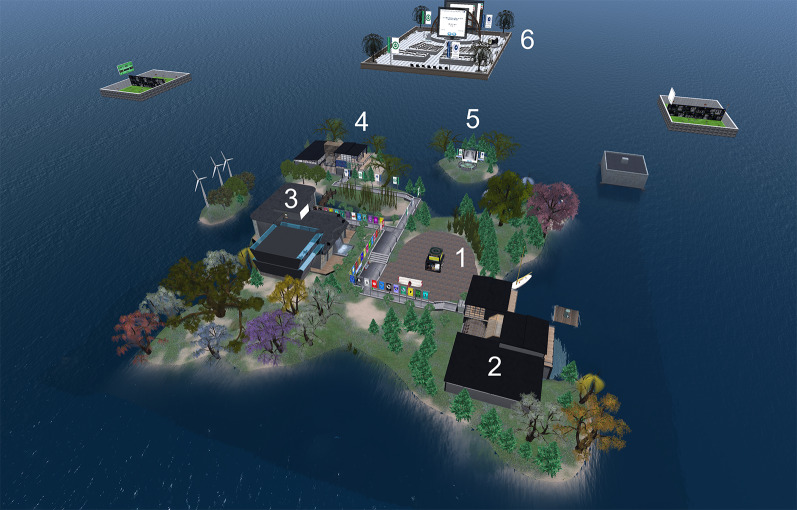
Aerial view of the Medical Master Island, in which the following are indicated: (1) the central esplanade, next to the arrival point; (2) the postgraduate building; (3) the Medical Master Conference Center; (4) the undergraduate building; (5) a small open-air auditorium, on an islet; and (6) a floating auditorium, in the air

The Medical Master Island reproduces a university campus, with a central esplanade surrounded by trees and walkways, educational buildings, open-air auditoriums (Fig. 1), and underwater settings (caves, palaces, submerged cellars, etc.) used to give a playful aspect to some learning activities.

### Participants

In February 2019, the game was introduced as a voluntary online activity for students enrolled for the first time in the four-month Radiology course. Students were informed that their participation would have no effect on their grades. They were invited to 2-h training sessions on Second Life, held on March. Several PDF files were provided with instructions for using Second Life and the rules of the game. Those students who wanted to participate in the game had to propose together with a partner, and the organisation randomly unified the pairs into teams of four. Participants were asked to submit their team name and colour, logo image, and choose a captain.

This study carried out within the framework of the Educational Innovation Project of the University of Malaga # PIE17-113 received the corresponding approval from the Vice-Rector's Office for Teaching and Research Staff. The students gave their explicit consent when they voluntarily agreed to participate. No additional ethical permission was needed.

### Structure and organisation of the game

The League of Rays team-based game was held from April 1 to May 19, 2019, organised in 6 stages of 7 days, from Monday to Sunday. Between the second and third stages, the game was interrupted for the Easter holidays. The first three stages were dedicated to radiological anatomy and the next three to radiological semiology. Thorax, abdomen, and musculoskeletal were treated successively in each 3-week block.

**Fig. 2:**
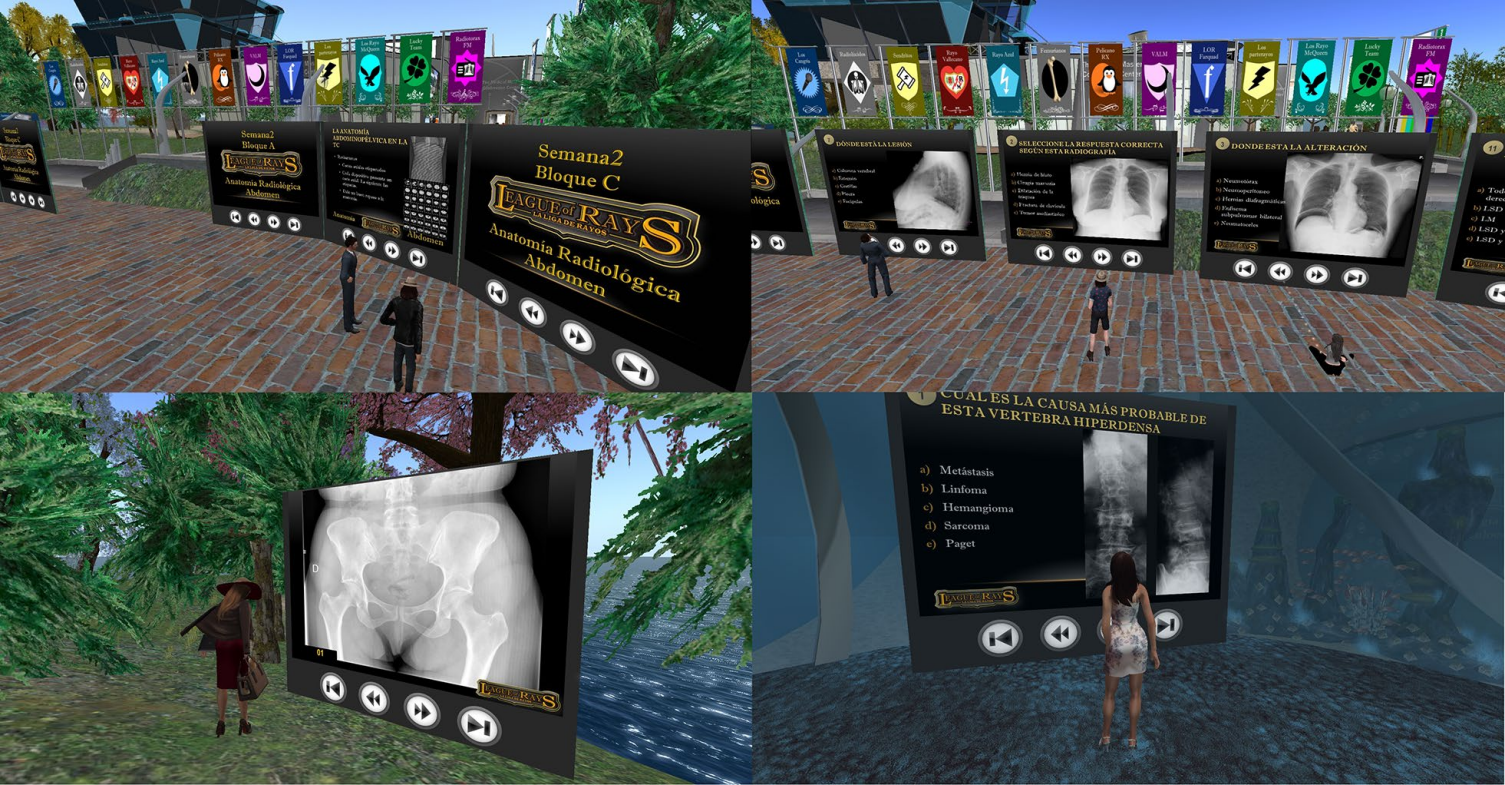
Different scenes during the League of Rays game. Top left: students viewing a set of three learning panels on radiographic anatomy of the abdomen. Top right: Students in front of panels with weekly tests on the central esplanade. Bottom left: a student in front of an N/P task located between the trees. Bottom right: student conducting a weekly test at an underwater facility

**Fig. 3:**
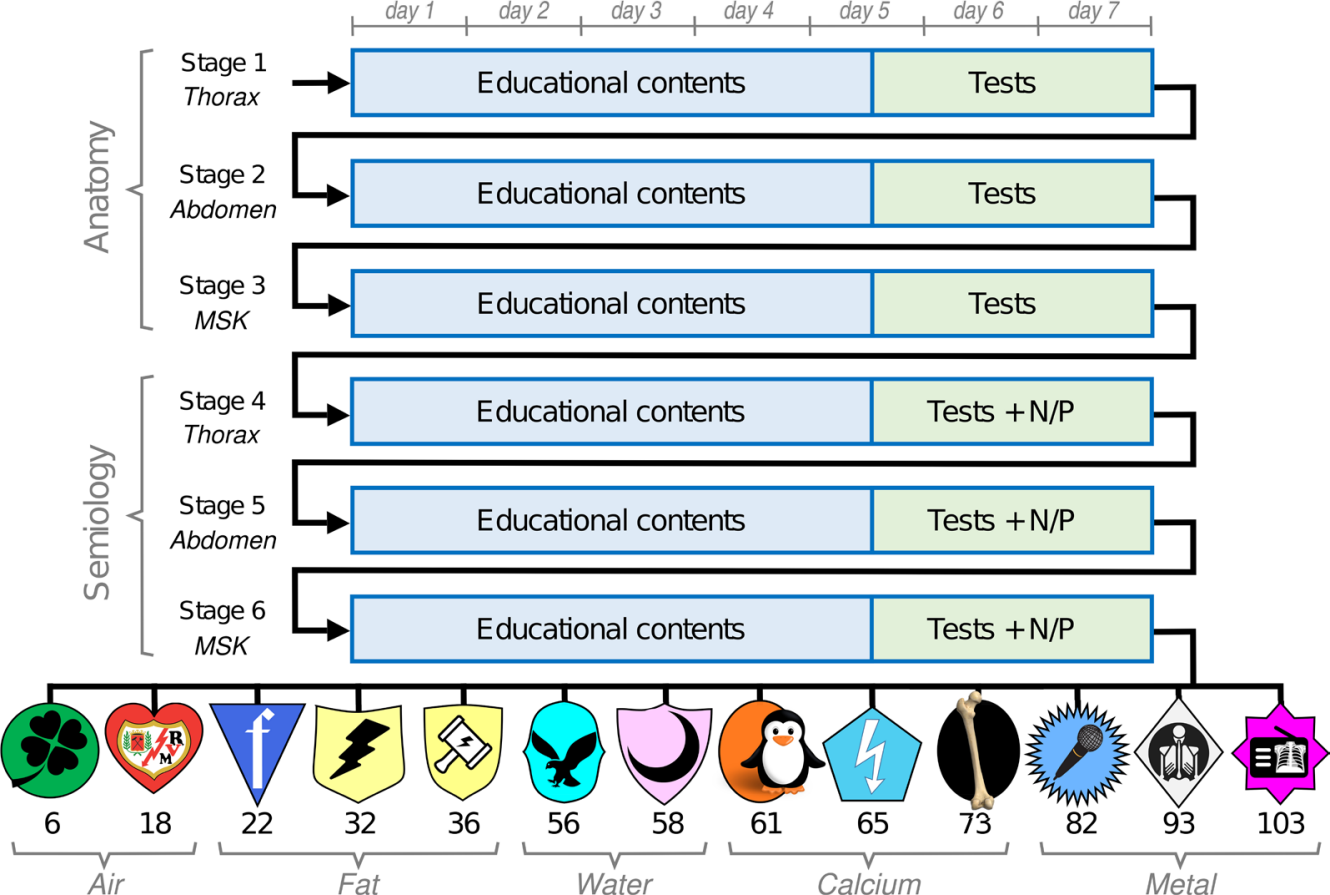
Diagram representing the flow of the Team-based League of Rays game. The 13 teams with the points achieved at the end of the competition are shown below, together with the badge that represents them. Test: Multi-choice test of 15 questions. N/P: Task "Normal or Pathological"

During the first 5 days of each stage, educational contents, elaborated from the syllabus and seminars of the course, and online resources with owned copyrights were presented in a set of 3 panels with 50 self-explanatory slides each. Each week, 6 sets of panels were arranged in the central esplanade of the island, available continuously for participants (Fig. 2). On the afternoon of the fifth day, the educational panels were replaced by single panels with 15 multi-choice questions. A database of 180 slides with multi-choice questions, 30 questions for each thematic block, was used for this purpose. Twelve test variants were elaborated, homogeneously distributing the 30 corresponding questions, so that each of them was in 6 test variants. Each week, a test variant was assigned to each participant, who had to answer it by sending a notecard to the teacher's avatar. Test variants were not repeated within the same team. Correct answers added one point and incorrect answers subtracted 0.25 points for each participant. The teams, ordered by the sum of their members' scores, received from 0 to 12 points according to their position. The points accumulated by each team determined the classification after each stage. The test panels were arranged in various places (among the trees, in the sky or under water), to give variability to the game (Fig. 2). In the last three stages, dedicated to semiology, an additional task called "Normal or Pathological" (N/P) was included, consisting of a panel with 20 radiographs, of which 12 were normal. The teams had to correctly diagnose pathological cases and identify normal ones and their captain had to send the notecard with the answers. Zero to 12 additional points were assigned to the teams, using the same methodology as in the individual tests. In this way, the maximum points achievable in the competition were 108 (12 points × 6 tests × 3 tasks). Figure 3 shows a complete diagram of the game to facilitate understanding. The evolution of the contest was publicised on the virtual campus (Moodle platform) of the Radiology course. All participants received a certificate, specifying their participation in an educational innovation project for 18 h.

### Assessment of the impact on learning

A 9-point Likert scale from 1 (very, very low mental effort) to 9 (very, very high mental effort) [29] was used to evaluate the cognitive load related to the review of the teaching content, the performance of the game tests, and the use of Second Life, at the end of the three blocks corresponding to anatomy and semiology. The test scores of each stage made it possible to measure the short-term knowledge profile of the participants. The correct answers were not provided during the game. One month after the game ended, all the students in the course were invited to a review seminar in the classroom, where the certificates and prizes of the participating teams were publicly given. The 180 questions of the game were reviewed except for 60 questions (10 randomly selected from each stage) that integrated a post-exposure test with 30 s to answer each question. Additionally, course scores were recorded to correlate with game scores. The results of participants and non-participants were compared.

### Evaluation of the experience

Students who participated in the game were asked to complete an experience evaluation questionnaire, based on that from previous studies [28], consisting of 23 statements with answers on a five-point Likert scale (7 about Second Life, 8 about the game, 4 about the presentation of the different stages and 4 about the multi-choice tests), an evaluation of 1 to 10 points of various aspects of the project and a text box to add open comments (See Additional file 1: Appendix 1). No personal identification was included in the evaluations, and they were analysed anonymously.

### Data analysis

Data were organised in Excel 2013 files (Microsoft, Redmond, WA, USA), and the SPSS statistical package, version 24 (IBM Corporation, Armonk, NY, USA), was used for statistical analysis. The Student’s t test for unpaired samples was used to assess the differences between participants and non-participants in the post-exposure test and course grades. Pearson's correlation coefficient was used to assess the correlation between the game tests with post-exposure tests and course grades. Statistical significance was accepted when a probability of error *p* < 0.05 was obtained.

To validate the questionnaire, Cronbach's α test was applied to the three constructs of the questionnaire: the student's experience in Second Life, the student's experience in the game, and the overall evaluation of the project. Kendall's τ-b test was used to evaluate the validity of the questions in each construct, indicating the agreement between the students when answering each item, with an accepted level of significance for *p* < 0.05. The adequacy of the analytical factor model was evaluated with the Kaiser–Meyer–Olkin (KMO) test.

The open comments included in the questionnaire were analysed through collaborative systematic coding by group consensus agreement [30], considering the codes obtained in previous studies on League of Rays [28]. In a first consensus meeting, four first-layer codes were established: positive, negative, suggestions, and team. The last, indicating that the comment made specific reference to teamwork. Second-layer subcodes were proposed and finally agreed upon in a second consensus meeting. The same comment could contain more than one different codes and/or subcodes.

## Results

### Participation and evolution of the game.

Fifty-two students out of 185 (28.1%), organised into 13 groups of 4 students, participated in the game. Table 1 shows the results of the weekly tests and the N/P tasks, together with the final score of each team. The correlation between the game score and the sum of accumulated correct answers for each team was excellent (Pearson coefficient = 0.973). There were 21 undelivered weekly tests and 4 undelivered N/P tasks.

**Table 1:** Results of the 13 participating teams, classified according to the final classification

Teams	Weekly tests			N/P tasks			Final points
	Hits^a^	(%)^a^	Test not delivered	Hits^b^	(%)^b^	Task not delivered	
Radiotorax FM	333	92.5	0	55	91.7	0	103
Radiolúcidos	297	82.5	0	54	90.0	0	93
Los Cangris	35	84.7	0	51	85.0	0	82
Femurianos	275	76.4	1	44	73.3	0	73
Rayo Azul	239	66.4	1	46	76.7	0	65
Pelicano RX	254	70.6	0	45	75.0	0	61
VALM	258	71.7	0	44	73.3	0	58
Los Rayo McQueen	278	77.2	1	24	40.0	1	56
Sendritos	210	58.3	1	40	66.7	0	36
Los Parterayos	215	59.7	1	41	68.3	0	32
LOR Farquad	176	48.9	5	31	51.7	0	22
Rayo Vallecano	149	41.4	4	29	48.3	0	18
Lucky Team	145	40.3	7	0	0.0	3	6

N/P: Normal/Pathological

^a^Number of correct answers for weekly tests on a maximum of 360 points (15 points × 6 weeks × 4 participants)

^b^Number of correct answers of the N/P tasks on a maximum of 60 points (20 points × 3 tasks)

### Impact on learning

The questions about cognitive load during the anatomy and semiology stages were answered by 46 (88.5%) and 31 (59.6%) participants, respectively. The mental effort to use Second Life was significantly lower during the first three weeks (cognitive load = 3.8 ± 2.5 vs 5.7 ± 2.0; *p* < 0.001). No significant differences were found in the mental effort involved in reviewing the teaching content (5.7 ± 1.1 vs 5.8 ± 1.5) or performing the tests (6.0 ± 1.8 vs 6.5 ± 1.6). The mean percentage of correct answers in the game tests was higher in the three stages of anatomy than in the three stages of semiology (75.5 ± 19.5 vs 68.5 ± 20.5; *p* = 0.009), although the lowest percentage of hits were obtained in thoracic anatomy (Table 2). The percentage of correct answers in the N/P tasks was 72.0 ± 17.0, with the lowest result in the chest tasks (Table 3).

**Table 2:** Correct questions in the tests each week (mean ± standard deviation) during the game, together with the number of tests not delivered

	Correct questions		Not delivered	
	*(mean ± standard deviation)*	(%)^a^	*N*	(%)^b^
Week 1. Thoracic anatomy	8.9 ± 2.9	59.6 ± 19.6	3	5.8
Week 2. Abdominal anatomy	12.3 ± 2.0	82.0 ± 13.4	5	9.6
Week 3 MSK anatomy	12.8 ± 2.1	85.3 ± 13.8	3	5.8
**ANATOMY**	**11.3 ± 2.9**	**75.5 ± 19.5**	**11**	**7.1**
Week 4. Thoracic semiology	10.4 ± 3.2	69.6 ± 21.4	5	9.6
Week 5. Abdominal semiology	10.8 ± 3.3	72.0 ± 21.9	3	5.8
Week 6. MSK semiology	9.6 ± 2.6	64.1 ± 17.7	2	3.8
**SEMIOLOGY**	**14.9 ± 2.5**	**68.5 ± 20.5**	**10**	**6.4**
**TOTAL**	**10.8 ± 3.0**	**72.0 ± 20.3**	**21**	**6.7**

Bold values indicate results of the three weeks of anatomy, the three weeks of semiology or the total of six weeks,respectively

^a^Percentage calculated on 15 questions for each test

^b^Percentage calculated with respect to 52 weekly tests

*N* Number of tests not delivered by students

**Table 3:** Correct questions in the "normal or pathological" tasks during the game, together with the number of tests and tasks not delivered

	Correct questions		Not delivered	
	*(mean ± standard deviation)*	(%)^a^	*N*	(%)^b^
Task N/P Chest	13.3 ± 3.7	66.7 ± 18.6	1	7.7
Task N/P Abdomen	15.0 ± 3.7	75.0 ± 18.7	1	7.7
Task N/P Musculoskeletal	14.9 ± 2.5	74.5 ± 24.7	2	15.4
**TOTAL**	**14.4 ± 3.4**	**72.0 ± 17.0**	**4**	**10.2**

Bold values indicate results of the total "normal or pathological" (N/P) tasks

^a^Percentage calculated on 20 questions for each task

^b^Percentage calculated with respect to 13 tasks of each anatomical location, 39 in total

*N* Number of tests not delivered by students

**Fig. 4:**
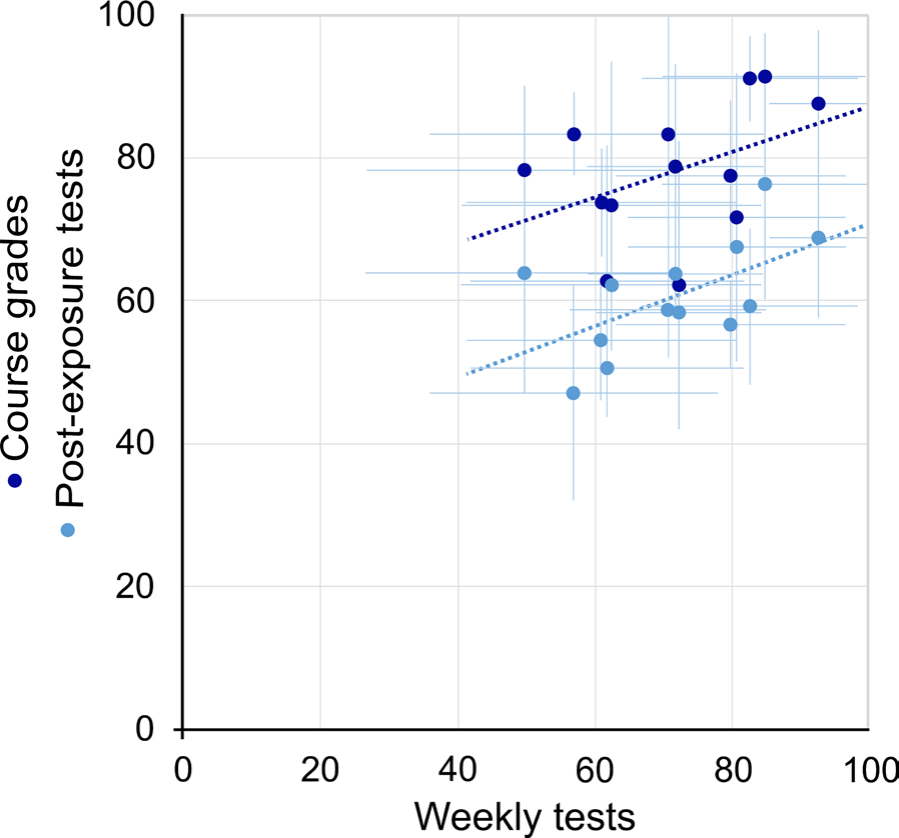
Graph showing the correlation of the results obtained by each team in the weekly game tests with the results obtained in the post-exposure tests (light blue points; Pearson's coefficient = 0.572) and with the course grades (dark blue points; Pearson's coefficient = 0.425). The data are the average scores obtained by teams, expressed as a percentage. Error bars represent the standard deviation

The post-exposure test was carried out by 45 participants and 83 non-participants. Participants obtained superior results than non-participants, with significant differences (*p* = 0.009) in the semiology questions (Table 4). The percentage of answers left blank for participants and non-participants was 2.4 ± 5.2 and 4.6 ± 6.7, respectively (*p* = 0.007). Forty-nine participants (94.2%) and 96 non-participants (72.1%) took the final examination in the June convocation, whose grades (mean percentage ± standard deviation) were 79.2 ± 15.3 and 71.3 ± 19.5, respectively (*p* = 0.021). The mean of correct answers by teams showed a low correlation with the final grades (Pearson's coefficient = 0.425) but moderate correlation with the post-exposure test (Pearson's coefficient = 0.572) (Fig. 4).

**Table 4:** Percentages of correct answers and answers left blank in the post-test (mean ± standard deviation) one month after the game

	Correct answers			Answers left balnk		
	Participants	Non-participants	*p*	Participants	Non-participants	*p*
Anatomy	69.6 ± 13.3	65.5 ± 11.7	0.094	0.4 ± 1.0	0.9 ± 1.7	0.056
Semiology	51.6 ± 13.5	40.8 ± 12.2	< 0.001	2.0 ± 2.5	3.7 ± 4.7	0.009
TOTAL	60.6 ± 11.9	53.2 ± 10.6	0.001	2.4 ± 3.2	4.6 ± 5.7	0.007

Participants (n = 45). Non-participants (n = 83)

### Students' perception

**Fig. 5:**
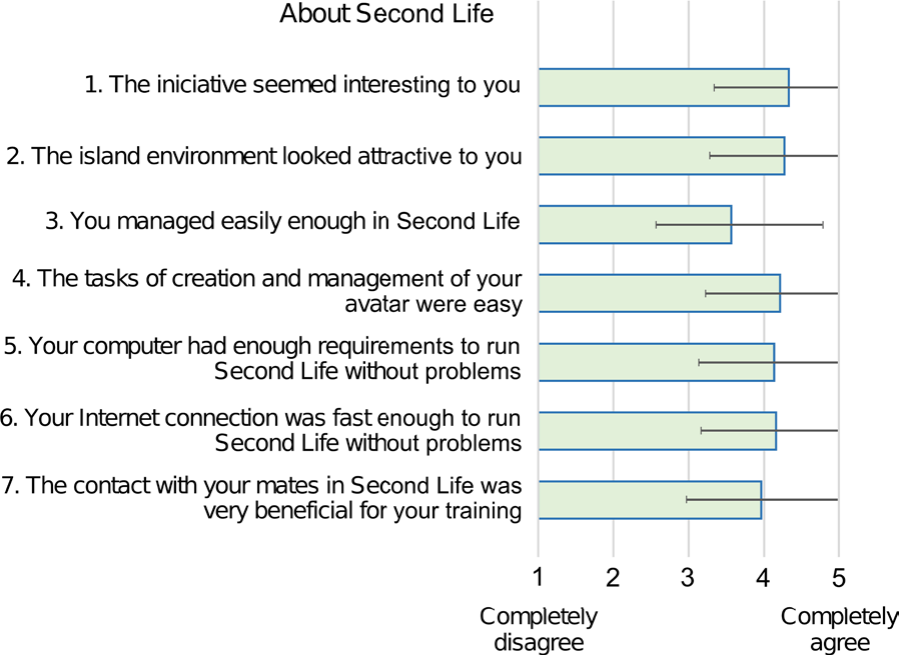
Bar diagram expressing the mean of the results of the evaluation questionnaire about Second Life on a five-point Likert scale. Error bars represent the standard deviation

**Fig. 6:**
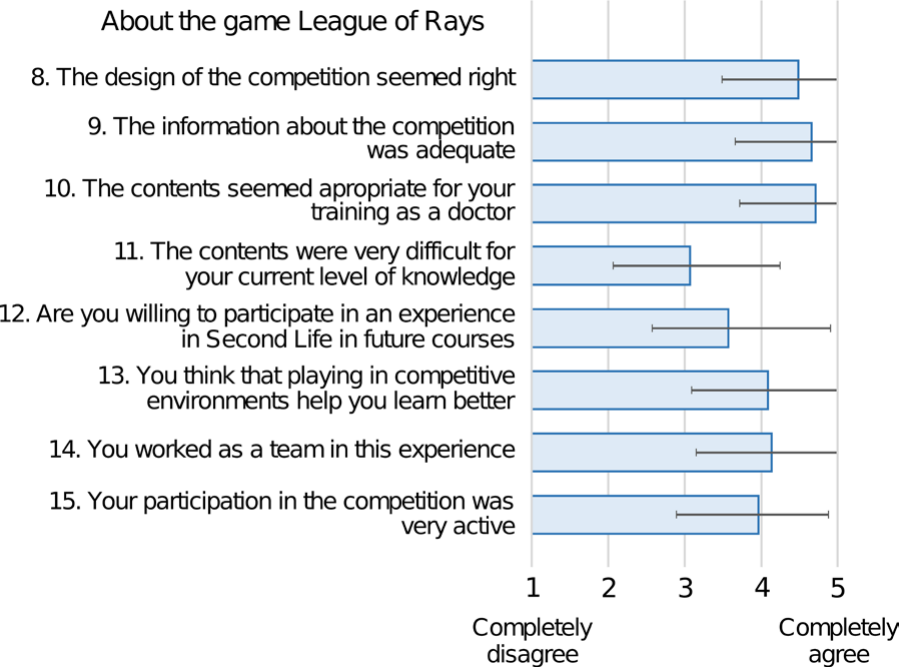
Bar diagram expressing the mean of the results of the evaluation questionnaire about the League of Rays game on a five-point Likert scale. Error bars represent the standard deviation

**Fig. 7:**
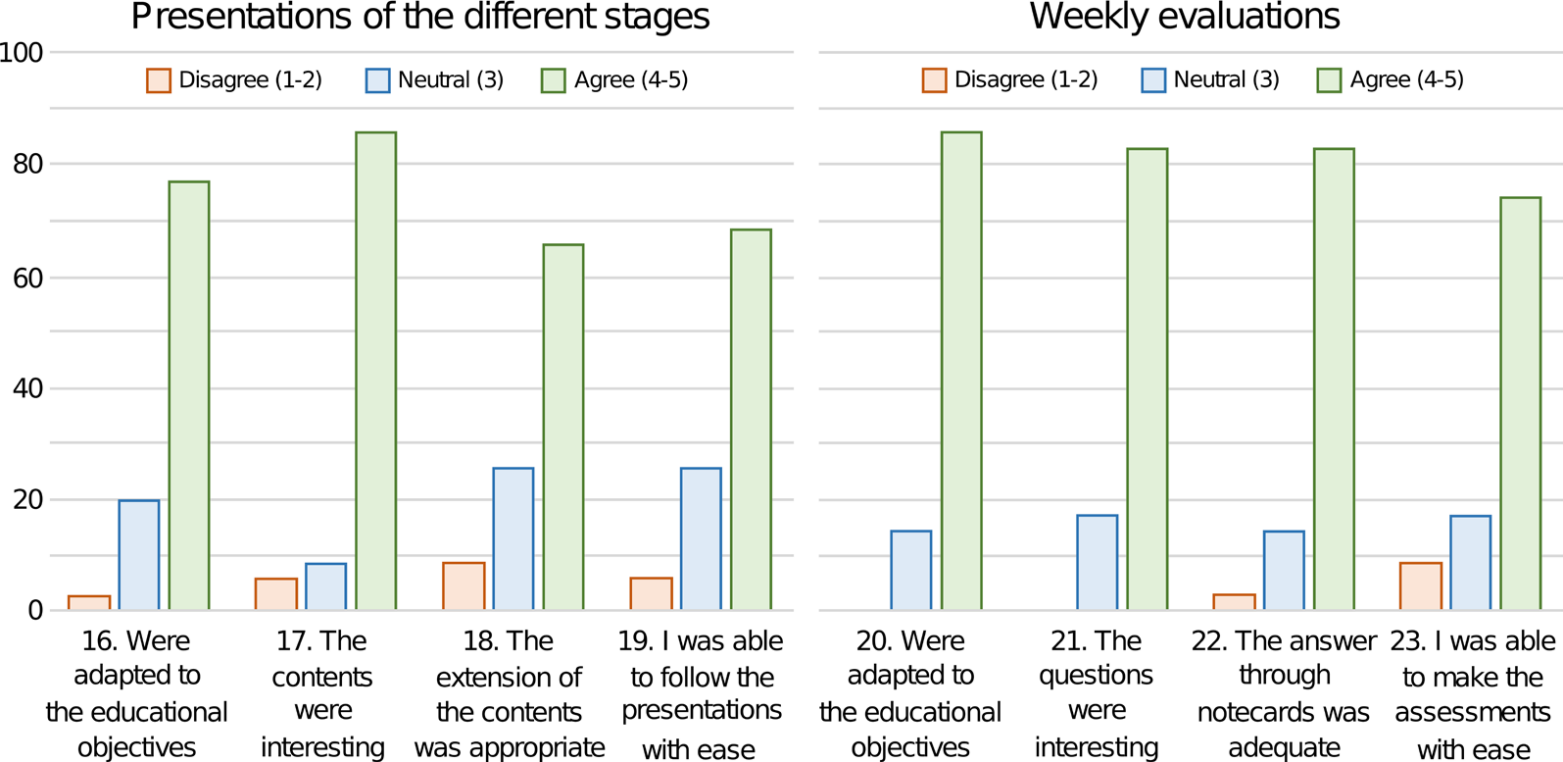
Bar diagram expressing the results of the evaluation questionnaire on the teaching presentations and the test-type evaluations of the game on a five-point Likert scale. The bars express the percentage of students who completely disagreed or disagreed (1–2), gave a neutral answer (3), or agree or completely agree (4–5)

Thirty-five participants (67.3%) completed the evaluation questionnaire. The questionnaire presented high internal consistency in the three constructs that comprise it: the students' experience in Second Life (Cronbach alpha = 0.83), the perception of the game (Cronbach α = 0.81), and the global evaluation of the project (Cronbach α = 0.95). Kendall's τ-b test correlation matrices can be seen in Additional file 1: Appendix 3. Its analysis revealed a positive correlation between all pairs of variables with respect to the evaluation of the global experience (τ-b > 0.41 *p* < 0.01) and with respect to participants' opinion about the experience in Second Life (τ-b > 0.25; *p* < 0.05 in 19/21 pairs combination). In exchange, the results were disparate regarding the perception of the students about the game, finding a positive correlation between variables 8, 9, and 10, related to the design, information, and the adequacy of the contents to medical training (τ-b > 0.34; *p* < 0.05). In the factor analysis of the questionnaire, the results indicated that the sampling adequacy for the items related to the students' experience in Second Life was moderate (KMO index = 0.743), for the items related to the students' perception of the game it was medium (KMO index = 0.612), and for the overall evaluation of the project it was excellent (KMO index = 0.902). The opinion of the students about Second Life was positive in general (mean values ≥ 4.0 ± 1.0 points on a five-point Likert scale), except for the statement *“you managed easily enough in Second Life”*, to which 8 students (23%) disagreed (responses 1–2), obtaining a mean value of 3.6 ± 1.2 (Fig. 5). The opinion of the students about the game (Fig. 6) was very positive in terms of design, information, and the adequacy of the contents for medical training (mean values ≥ 4.5 ± 0.7). The participants agreed (answers 4–5) in having worked as a team during the game and in learning better in competitive games by 83% and 74% , respectively. There was some dispersion in the answers about the difficulty of the contents, 34% agreed that they were difficult, 23% gave a neutral answer (3), and 43% did not find them difficult. Most of the students found the educational presentations and the evaluations during the game interesting, appropriate to the learning objectives, adequate, and easy to follow or perform (Fig. 7). Finally, the qualification of the experience, from 1 to 10 points, obtained very positive values (mean values ≥ 7.8), highlighting the teacher, the educational content, and the usefulness for their training (Table 5).

**Table 5:** Results of the global evaluation of the experience

Items	Scores
The experience globally	8.1 ± 1.3
The organisation of the project	8.6 ± 1.2
The environment of the island	8.6 ± 1.7
Educational content	8.8 ± 1.4
The usefulness for your education	8.7 ± 1.4
The teacher	9.3 ± 1.1
Interaction with peers	7.8 ± 2.0
The presentations	8.4 ± 1.4
The evaluations	8.2 ± 1.3
Connectivity to Second Life	7.9 ± 1.9

The results represent the mean and standard deviation of 35 questionnaires for each item on a scale of 1 to 10

Fourteen questionnaires (40%) included open comments (see Additional file 1: Appendix 2). Ten of them included **positive** comments; 8 were subcoded as **appreciation**, since they included expressions such as *"great initiative"*, *"very interesting"*, or *"highly recommended"*; 7 reflected aspects related to the **formative** nature of the experience, with expressions such as *“I have learned a lot”, “great help to practice with cases'' or “it has helped me to understand radiology”*; 5 were subcoded as **fun**, as students found it an entertaining or fun learning method. Other positive comments included words of **thanks**, **availability** to participate in future editions, and **motivation**, expressly indicating this aspect of the experience. Some participants included several positive subcodes in their comment, for example: *“I think that learning through competition is something that young people are very interested in. Generally, we do not like to lose, especially if it is against friends who we are going to see the next day in class. I think it is a very fun way to keep track of the subject and stay motivated throughout the semester"*. Five questionnaires included comments coded as **negative**. On four occasions, referred to the game's **schedule**, indicating that it ended near the examination period. Other negative subcodes were **technical limitation** to handle Second Life, **content difficulty**, **lack of time** due to having a lot of work with other courses. Comments coded as **suggestions** were found in eight questionnaires. Four of them proposing **rules of the game**, some almost describe a completely different game, others suggest an earlier **schedule**. Finally, there were four comments coded as **team**: **team-choice**, indicating that teams should be chosen by the members; **team-demand**, referring to the fact that more demand for participation should be requested from the teams; **team-help**, expressing the help provided by their teammates to solve technical problems; or **team-inconvenience**, verbatim as follows: *“… team play is somewhat more cumbersome than individual participation, but in the end the result has been good."*

## Discussion

Today's medical students, young adults of Generation Z, make extensive use of new media technologies [31], enjoy learning through them [32], and often use virtual platform games for entertainment [33]. Game-based radiology education is an exciting and innovative teaching method with numerous benefits, such as student engagement, social interaction, instant feedback, and a personalised learning environment [34]. The virtual world Second Life provides interesting perspectives to play simulation games with proven educational success [35–37]. Although the League of Rays is not a medical simulation game, but a contest developed on an imaginary island, which provides learning reinforcement on basic radiological anatomy and semiology. It has been well valued by medical students competing individually [28] and also by teams, as this study shows, with great recognition for the design and organisation of the contest, the educational content and the adaptation to their medical training. This team competition could be considered as a mandatory activity of the course, but when individual participation in League of Rays is mandatory, the acceptance of virtual world technology decreases, the opinion about the game worsens, and the average score in the game decreases (unpublished observations of the authors).

Radiology blended learning formats that include new pedagogical concepts and current technologies allow improving the performance, satisfaction, and engagement of medical students [38]. The League of Rays game provides educational contents devised to be both a review and a training complement to the formal Radiology course, in an attractive context, of active and dynamic participation. In addition, it allows students to know the strengths and weaknesses of their knowledge, a recognised value of digital games [2]. For example, the worst results of tests and game tasks were obtained in the thoracic stages. As chest radiology is essential in medical practice [39] and especially difficult to interpret due to overlapping anatomical structures [40], reinforcement of thoracic radiology learning is especially helpful.

The present study has shown that team participation in the League of Rays is feasible with a simple adaptation of the game rules, allowing teams to be classified with a scoring system highly correlated with the number of correct answers. Games in which teams of students compete are considered cooperative learning techniques that combine group rewards with individual responsibility, that is, collaboration with competition [7]. In the League of Rays, participants are encouraged to be responsible in their collective work, because if any of them get few hits or do not take the test, it will mean a decrease in their team in the classification. Success in obtaining the cooperation and commitment of the students depends on them, the game designers, and the educators [7]. Eighty-three percent of participants in this study agreed that they worked as a team. The motivation to participate in teams is perfectly reflected in one of the open comments: *“… When knowledge is associated with positive experiences such as winning as a team or putting class theory into a game, everything becomes less serious and gives more ground to take this activity as something relaxed and entertaining"*. Other motivating elements for the participants in this study are the recognition (and good evaluation) of the design and organisation of the contest and the identification of the educational contents as a suitable learning reinforcement for their medical education. It would be interesting to follow these students to determine the possible influence on their choice of radiology as a medical specialty.

Competitive learning used to be associated with the traditional classroom and the competitive behaviour of students, being a subject of criticism [41]. Today, although it remains a topic of debate, competitive online learning through digital games is a powerful tool that can lead to favourable academic results [42]. Participants in the League of Rays performed better in post-exposure tests than non-participants (*p* < 0.007), especially at the expense of semiology questions. In addition, they obtained better final grades than their peers (*p* < 0.021). Both facts can be justified by an impact of the game on radiology learning or by the participation of a sector of students that is more active and motivated than the rest. Team results in the game showed moderate correlation with post-exposure tests but low correlation with course grades. The latter is reasonable, since the course has additional contents to those dealt with in the game (neuroimaging, head and neck, ultrasound, nuclear medicine, etc.) and all participants had access to formal teaching activities to prepare for the examination.

This study has several limitations. One is the possible demotivation and abandonment of participation in the game. The last three teams in the classification accumulated 76.2% of the undelivered weekly tests, and the last team did not turn in any of the N/P tasks. Perhaps it is better for students to propose all four members of their team rather than assigning pairs of students. It would be interesting to deepen the way in which students experience competition, since they are exposed to messages of social comparison that can influence their self-conception, emotions and actions [43], and pressure and anxiety about time spent playing, in conflict with other academic tasks, can inhibit participation and collaboration [44]. It would also be interesting to evaluate in depth the feeling of cooperation given and received, since the inhibition of a partner can modify the motivation of the team, or the role of leadership or mentor can modulate the cognitive process of their colleagues [45].

Technical limitations have been described as a factor that limits student participation in Second Life teaching activities [46, 47]. In other studies, on teaching radiology in Second Life to medical students, 9–11% of these expressed serious technical limitations [25, 27, 28]. In the present study, only one student reported on technical limitations and was able to participate in the game thanks to the help of his/her classmates, which expresses the collaborative nature of this edition by teams.

This study does not assess radiological anatomy and radiological semiology of neuroradiology, breast radiology, or vascular interventional radiology, which could be included in future activities of this project. One of the future perspectives of this project is to reproduce it with students from different universities since the adaptation of the rules of the game allows for an inter-university competition. This would reduce the proximity bias to analyse the perception of the participants, since in this study the positive evaluation of the experience could be influenced in part because it is conducted by the professor responsible for their formal Radiology course. Furthermore, the sense of belonging, competing with others at the national level, can have very motivating results, as other educational competitions with medical students have shown [48]. Competition with students from various medical schools could reach interesting heights. In a further step, cooperation with international universities would allow evaluating the results and limitations of the learning game in an international and multicultural setting.

## Conclusion

A multi-user game to learn radiology in Second Life adapted to team competition has been very positively perceived by third-year medical students, who highly valued its content, organisation, and usefulness for their training. Most of the participants agreed that they had collaborated as a team and that playing in competitive environments helps them learn better. The best post-exposure and academic results compared to non-participating students indicate the potential impact of the game on learning. This study lays the necessary foundations to repeat the competition with students from different medical schools.

## Supplementary Information

**Additional file 1**. **Appendix 1.** Questionnaire. **Appendix 2.** Open-ended comments. **Appendix 3.** Correlation matrices of the questionnaire.

## Data Availability

The datasets used and/or analysed during the current study are available from the corresponding author on reasonable request.

## Abbreviations

N/P: Normal or pathological
PDF: Portable document format
