# Supplementary material for: A team-based competition for undergraduate medical students to learn radiology within the virtual world Second Life
Source: Insights Imaging. 2021 Jun 29;12:89. doi: 10.1186/s13244-021-01032-3 (PMC8241985; doi:10.1186/s13244-021-01032-3)
Supplement: Supplementary file 1 — Additional file 1. Appendix 1. Questionnaire. Appendix 2. Open-ended comments. Appendix 3. Correlation matrices of the questionnaire. [file 13244_2021_1032_MOESM1_ESM.pdf]

Appendix 1. QUESTIONNAIRE

Please, answer the following statements inside the box, scoring from 1 to 5 (1: completely disagree, 5: completely agree).

About Second Life

1. Learning radiology in Second Life seemed interesting to you................................... ☐
2. The island environment looked attractive to you .... ☐
3. You managed easily enough inside Second Life.. ☐
4. The tasks of creation and management of your avatar were easy............................ ☐
5. Your computer had enough requirements to run Second Life without problems......... ☐
6. Your Internet connection was fast enough to run Second Life without problems .... ☐
7. The contact with your mates in Second Life was very beneficial for your training....... ☐

About League of Rays

8. The design of the competition seemed right to you .... ☐
9. The information about the competition was adequate .... ☐
10. The contents seemed appropriate for your training as a doctor................................ ☐
11. The contents were very difficult for your current level of knowledge.......................... ☐
12. Are you willing to participate in an experience in Second Life in future courses .... ☐
13. You think that playing in competitive environments help you learn better.................. ☐
14. You worked as a team in this experience... ☐
15. Your participation in the competition was very active .... ☐

Presentations of the different stages

16. The presentations were adapted to the educational objectives .... ☐
17. The contents of the presentations were interesting .... ☐
18. The extension of the contents was adequate .... ☐
19. You were able to follow the presentations with ease .... ☐

Weekly evaluations

20. The questions were adapted to the educational objectives .... ☐
21. The question were interesting .... ☐
22. The response through notecards was adequate .... ☐
23. You were able to make the assessments with ease .... ☐

***Appendix 1*. QUESTIONNAIRE**

Important! Qualification from 1 to 10

To finalize, please qualify inside the box scoring from 1 to 10 the following aspects

The experience globally .... ☐

The organization of the project............................ ☐

The island environment.. ☐

The educational contents..................................... ☐

The usefulness for your education .... ☐

The professor .... ☐

The interaction with your mates .... ☐

The presentations of each stage......................... ☐

The evaluations .... ☐

The connectivity to Second Life .... ☐

Free Comments

If you want to add anything, please use the next textbox

BLINDED 2019

Appendix 2. OPEN-ENDED COMMENTS

Questionnaire #01: A great initiative. I have learned a lot (I hope, we will see the exam ha ha).

Questionnaire #02: The only thing that would change would be the chosen time period and the game mode for this year. *BLINDED* would have started earlier, because it has reached the end of May. Also, team play is somewhat more cumbersome than individual participation, but in the end the result has been good. Very good initiative and a great help to prepare and practice with cases

Questionnaire #04: *No comments.*

Questionnaire #05: I think it would have been better to have started the competition earlier and finished before the exams.

Questionnaire #06: I appreciate the interest and dedication in looking for alternatives to the traditional teaching model, and especially, healthy competition and good vibes.

Questionnaire #08: *No comments.*

Questionnaire #09: The groups should be able to be chosen by the members, not just the couple with whom you sign up, because in the end if they are people with whom you do not get along at work, you tend to individualize the tests and do things separately. Take into account specific dates such as Easter. I would suggest that the contents be posted on the island even when the evaluation tests are done, because it makes you be more attentive and better search for references, because in the end when we do the evaluation we look at it online, and if we had the contents on the island we would look for them again to check.

Questionnaire #10: *No comments.*

Questionnaire #11: *No comments.*

Questionnaire #13: *No comments.*

Questionnaire #14: It has been a good way of working the course on a weekly basis without involving a great effort, I can say that I have had fun learning radiology. It is a very interesting and fun project, thank you for your involvement and for finding such a great didactic method.

Questionnaire #15: *No comments.*

Questionnaire #16 An interesting idea would be to hold an Olympics in small groups (about 20-30 people per group), with a specific theme for each of them. In this way, the components of each subgroup would prepare these topics and compete live. I think it's a good method, since it would

force the members to prepare globally for their theme. With this we would prevent each person in your house from reading what is in front of them and we would gain attention and dynamism.

Questionnaire #17: *No comments.*

Questionnaire #19: *No comments.*

Questionnaire #22: The normal or pathological tasks along with the week tests were too dense. It could have started earlier so it would have given time for the N / P cases to be in different weeks. I learned a lot in the *BLINDED*, highly recommended.

Questionnaire #24: *No comments.*

Questionnaire #25: *No comments.*

Questionnaire #26: *No comments.*

Questionnaire #27: *No comments.*

Questionnaire #30: The only thing I would add would be the answers to the tests so that each student had the possibility to see where they failed.

Questionnaire #31: *No comments.*

Questionnaire #32: *No comments.*

Questionnaire #33: It has helped me a lot to understand radiology in a very fun way, at first I was very lost with the course, it was difficult for me to see the pathologies in the x-rays and especially in the CT, but with the slides that were presented before the tests and with the tests themselves, it has helped me a lot to understand everything better. Very interesting this experience!

Questionnaire #34: *No comments.*

Questionnaire #37: I have not been able to get the application to work properly. Throughout the *BLINDED*, I have needed my teammates to pass me the link to see the presentations. Still, I think it is a very fun and educational way to learn radiology, so I would like to be able to solve the problems I have had to participate again next time.

Questionnaire #38: *No comments.*

Questionnaire #39: *No comments.*

Questionnaire #41: *No comments.*

Questionnaire #45: *No comments.*

Questionnaire #46: *No comments.*

Questionnaire #47: The overview of the *BLINDED* is great because we have learned in the best way that can be learned: through positive stimuli (although it sounds a bit like Paulov's dog). When knowledge is associated with positive experiences such as winning as a team or putting classroom theory into a game, everything becomes less serious and gives more ground to take this activity as something relaxed and entertaining, in short, at least I have learned, because now things sound much more familiar to me and I had no fear of losing or winning.

I think there should be a little more demand from the groups (although I was last with my group) that is, put a minimum of "tranquility". Said in Christian, an alternative would be to eliminate the groups and for the winner to be the only one left on the island, so people would strive to do better, the bad thing is that not everyone could do tests of all the matter because they would disqualified, it would be necessary to do sub-competitions of repechage, in any way, I have found *BLINDED* a success because you really learn. It is also perhaps a good time to thank the teacher for his dedication to the classes and his willingness to teach us with enthusiasm and closeness.

Questionnaire #48: Although the experience has been great, I have not been able to follow the competition 100% because, coming from another university (transfer of file), I have as many second-year subjects, therefore, I would like to be able to participate next year.

Questionnaire #50: *No comments.*

Questionnaire #52: I think that learning through competition is something that young people are very interested in. Generally we do not like to lose, and more if it is against friends that we are going to see the next day in class. I think it is a very fun way to keep track of the course and stay motivated throughout the semester.

What I would propose would be a compensation in the grades of the course, because it really supposes a time of the week that you take away from other things. For example: Each week the team that wins the day earns 0.1 points that are added extra to the final grade. This would mean that no team falls off the hook, even if it is the last in the classification. It is just an example.

Appendix 3. Correlation matrices of the questionnaire.

Kendal Tau-b correlation matrix of variables 01 to 07 of the questionnaire. Construct: The students' experience in Second Life.

		VAR01	VAR02	VAR03	VAR04	VAR05	VAR06	VAR07
VAR01. The iniciative seemed interesting to you	R	1.000						
	P	.						
VAR02. The island envirnoment looked attractive to you	R	0.575*	1.000					
	P	<0.001	.					
VAR03. You managed easily enoug in Second Life	R	0.394*	0.484*	1.000				
	P	0.008	0.001	.				
VAR04. The tasks of creation and management of your avatar were easy	R	0.405*	0.299	0.462*	1.000			
	P	0.009	0.051	0.002	.			
VAR05. Your computer had enough requirements to run Second Life without problems	R	0.258	0.306	0.408*	0.452*	1.000		
	P	0.096	0.046	0.006	0.003	.		
VAR06. Your Internet connection was fast enough to run Second Life without problems	R	0.290	0.305	0.433*	0.349	0.603*	1.000	
	P	0.059	0.045	0.003	0.022	<0.001	.	
VAR07. The contact with your mates in Second Life was very beneficial for your training	R	0.560*	0.559*	0.353	0.389	0.351	0.265	1.000
	P	<0.001	<0.001	0.016	0.010	0.021	0.079	.

N = 35. R: correlation coefficient. P. Probability of significance (bilateral). Cells in green colour mean that the correlation is significant at P<0.05 level. *: The correlation is significant at P<0.01 level.

Appendix 3. Correlation matrices of the questionnaire.

Kendal Tau-b correlation matrix of variables 08 to 23 of the questionnaire. Construct: The students' perception of the game. (1/2)

		VAR08	VAR09	VAR10	VAR11	VAR12	VAR13	VAR14	VAR15	VAR16	VAR17	VAR18	VAR19	VAR20	VAR21	VAR22	VAR23
VAR08. The design of the competition seemed right	*R*	1.000															
	P	.															
VAR09. The information about the competition was adequate	*R*	0.335	1.000														
	P	0.045	.														
VAR10. The contents seemed appropriate for your training as a doctor	*R*	0.415*	0.408	1.000													
	P	0.012	0.014	.													
VAR11. The contents were very difficult for your level of knowledge	*R*	0.107	-0.059	-0.129	1.000												
	P	0.487	0.701	0.397	.												
VAR12. Are you willing to participate in an experience in Second Life in future courses	*R*	-0.008	0.371	0.339	-0.164	1.000											
	P	0.959	0.016	0.026	0.245	.											
VAR13. You think that playing in competitive environments help you learn better	*R*	0.008	-0.107	0.226	-0.039	0.363	1.000										
	P	0.958	0.499	0.152	0.789	0.013	.										
VAR14. You worked as a team in this experience	*R*	0.181	0.232	0.484*	-0.073	0.117	0.137	1.000									
	P	0.257	0.146	0.002	0.620	0.423	0.366	.									
VAR15. Your participation in the competition was very active	*R*	0.157	-0.041	0.323	0.085	-0.234	0.172	0.268	1.000								
	P	0.319	0.795	0.038	0.555	0.106	0.251	0.074	.								

N = 35. *R*: correlation coefficient. *P*. Probability of significance (bilateral). Cells in green colour mean that the correlation is significant at P<0.05 level. *: The correlation is significant at P<0.01 level.

Appendix 3. Correlation matrices of the questionnaire.

Kendal Tau-b correlation matrix of variables 08 to 23 of the questionnaire. Construct: The students' perception of the game. (2/2)

VAR16. The presentations were adapted to the educational objectives	*R*	0.154	0.554*	0.395	0.036	0.265	-0.141	0.264	0.065	1.000							
	P	0.339	0.001	0.013	0.807	0.073	0.357	0.085	0.667	.							
VAR17. The contents of the presentations were interesting	*R*	0.175	0.387	0.231	0.000	-0.009	-0.214	0.326	0.056	0.701*	1.000						
	P	0.272	0.015	0.145	1.000	0.949	0.158	0.032	0.709	<0.001	.						
VAR18. The extension of the contents was adequate	*R*	-0.043	0.384	0.333	-0.331	0.240	-0.106	0.333	0.092	0.618*	0.521*	1.000					
	P	0.784	0.014	0.032	0.021	0.094	0.474	0.025	0.530	<0.001	<0.001	.					
VAR19. You were able to follow the presentations with ease	*R*	0.014	0.392	0.343	-0.331	-0.002	-0.194	0.345	0.187	0.425*	0.567*	0.603*	1.000				
	P	0.931	0.013	0.028	0.022	0.988	0.195	0.022	0.206	0.005	<0.001	<0.001	.				
VAR20. The questions were adapted to the educational objectives	*R*	0.340	0.263	0.559*	-0.114	0.227	0.148	0.449*	0.456*	0.323	0.095	0.196	0.241	1.000			
	P	0.035	0.104	<0.001	0.441	0.127	0.337	0.004	0.003	0.038	0.537	0.196	0.113	.			
VAR21. The questions were interesting	*R*	0.347	0.150	0.503*	0.173	0.224	0.335	0.376	0.110	0.225	0.198	0.106	0.140	0.455*	1.000		
	P	0.032	0.354	0.002	0.245	0.133	0.030	0.015	0.471	0.149	0.200	0.482	0.357	0.004	.		
VAR22. The response through notecards was adequate	*R*	0.172	0.108	0.311	0.064	0.214	0.202	0.333	0.217	0.183	0.131	0.015	0.077	0.479*	0.473*	1.000	
	P	0.285	0.501	0.051	0.664	0.147	0.186	0.030	0.150	0.237	0.391	0.922	0.611	0.002	0.002	.	
VAR23. You were able to make the assessments with ease	*R*	0.138	0.059	0.221	-0.171	-0.064	-0.010	0.352	0.202	0.145	0.305	0.092	0.374	0.363	0.241	0.482*	1.000
	P	0.382	0.706	0.156	0.237	0.658	0.948	0.019	0.173	0.338	0.042	0.533	0.012	0.017	0.113	0.001	.
		**VAR08**	**VAR09**	**VAR10**	**VAR11**	**VAR12**	**VAR13**	**VAR14**	**VAR15**	**VAR16**	**VAR17**	**VAR18**	**VAR19**	**VAR20**	**VAR21**	**VAR22**	**VAR23**

N = 35. *R*: correlation coefficient. *P*. Probability of significance (bilateral). Cells in green colour mean that the correlation is significant at P<0.05 level. *: The correlation is significant at P<0.01 level.

Appendix 3. Correlation matrices of the questionnaire.

Kendal Tau-b correlation matrix of variables 24 to 33 of the questionnaire. Construct: Global evaluation of the project.

		VAR24	VAR25	VAR26	VAR27	VAR28	VAR29	VAR30	VAR31	VAR32	VAR33
VAR24. The experience globally	*R*	1.000									
	P	.									
VAR25. The organization of the project	*R*	0.668*	1.000								
	P	<0.001	.								
VAR26. The island environment	*R*	0.561*	0.625*	1.000							
	P	<0.001	<0.001	.							
VAR27. The educational contents	*R*	0.584*	0.597*	0.652*	1.000						
	P	<0.001	<0.001	<0.001	.						
VAR28. The usefulness for your education	*R*	0.647*	0.499*	0.595*	0.624*	1.000					
	P	<0.001	0.001	<0.001	<0.001	.					
VAR29. The professor	*R*	0.602*	0.601*	0.640*	0.766*	0.585*	1.000				
	P	<0.001	<0.001	<0.001	<0.001	<0.001	.				
VAR30. The interaction with your mates	*R*	0.653*	0.511*	0.616*	0.599*	0.630*	0.524*	1.000			
	P	<0.001	<0.001	<0.001	<0.001	<0.001	<0.001	.			
VAR31. The presentation of each stage	*R*	0.532*	0.584*	0.590*	0.637*	0.507*	0.573*	0.577*	1.000		
	P	<0.001	<0.001	<0.001	<0.001	<0.001	<0.001	<0.001	.		
VAR32. The evaluations	*R*	0.428*	0.435*	0.463*	0.510*	0.445*	0.413*	0.504*	0.692*	1.000	
	P	0.002	0.002	0.001	<0.001	0.002	0.005	<0.001	<0.001	.	
VAR33. The connectivity to Second Life	*R*	0.459*	0.525*	0.584*	0.531*	0.423*	0.499*	0.452*	0.478*	0.547*	1.000
	P	0.001	<0.001	<0.001	<0.001	0.003	0.001	0.001	0.001	<0.001	.

N = 35. *R*: correlation coefficient. *P*. Probability of significance (bilateral). Cells in green colour mean that the correlation is significant at P<0.05 level. *: The correlation is significant at P<0.01 level.
